# Metabolic syndrome among Sri Lankan adults: prevalence, patterns and correlates

**DOI:** 10.1186/1758-5996-4-24

**Published:** 2012-05-31

**Authors:** Prasad Katulanda, Priyanga Ranasinghe, Ranil Jayawardana, Rezvi Sheriff, David R Matthews

**Affiliations:** 1 Diabetes Research Unit, Department of Clinical Medicine, Faculty of Medicine, University of Colombo, Colombo, Sri Lanka; 2 Oxford Centre for Diabetes, Endocrinology and Metabolism, University of Oxford, Oxford, UK; 3 Department of Pharmacology, Faculty of Medicine, University of Colombo, Colombo, Sri Lanka; 4 Institute of Health and Biomedical Innovation, Queensland University of Technology, Brisbane, Queensland, Australia

**Keywords:** Diabetes mellitus, Metabolic syndrome, Prevalence, Sri Lanka, Developing country, South Asia

## Abstract

Metabolic Syndrome (MS) increases the risk for Coronary Artery Disease, stroke and diabetes. MS is twice more common amongst South Asian immigrants in US compared to native Caucasians. There are no nationally representative studies on prevalence of MS from any of the South Asian countries. The present study aims to evaluate the prevalence of MS among Sri Lankan adults and investigates its relationships with socio-demographic, clinical and biochemical parameters. Data on MS and its associated details were obtained from a population-based cross-sectional study conducted between years 2005–2006. MS was defined according to the International Diabetes Federation criteria. A binary logistic regression analysis was performed using the dichotomous variable MS (0 = absent, 1 = present). The independent co-variants were: gender, age category, area of residence, ethnicity, level of education, income and physical activity. Sample size was 4,485 (Response rate–89.7%), 39.5% were males and mean age was 46.1 ± 15.1 years. The crude prevalence of MS was 27.1% (95% CI: 25.8–28.5), and age-adjusted prevalence was 24.3% (95% CI: 23.0–25.6). Prevalence in males and females were 18.4% (95% CI: 16.5–20.3) and 28.3% (95% CI: 26.6–30.0) respectively (p < 0.001). Urban adults (34.8% [95% CI: 31.8–37.9]) had a significantly higher prevalence than rural adults (21.6% [95% CI: 20.2–23.0]). Among ethnic groups, the highest prevalence of MS was observed in Sri Lankan Moors (43.0% [95% CI: 37.2–48.9]). In all adults, MS was observed in those with the highest level of education and monthly household income. Prevalence of MS in the different physical activity categories of the IPAQ were; ‘inactive’–38.8% (95% CI 34.5-43.2), ‘moderately active’–33.5% (95% CI 30.9-36.1) and ‘active’–21.1% (95% CI 19.6-22.7). The results of the binary logistic regression analysis indicates that female gender (OR:1.7), increasing age, urban living (OR:1.7), Moor ethnicity (OR:2.6), secondary (OR:1.5) and tertiary levels of education (OR:2.3), monthly household income LKR 7,000–24,999 (OR:1.5) and >50,000 (OR:2.1), and physical inactivity (OR:1.6), all significantly increased risk of developing MS. MS is common among Sri Lankan adults affecting nearly one-fourth of the population. Female gender, increasing age, urban living, higher socio-economical status and physical inactivity were important associated factors.

## Introduction

Metabolic Syndrome (also known as cardio-metabolic syndrome, syndrome X and insulin resistance syndrome) is a name given to a group of risk factors when occurring together increases the risk for Coronary Artery Disease (CAD), stroke, and type-2 diabetes [1,2]. These risk factors include; obesity, dysglycaemia, dyslipidaemia and hypertension [3]. Insulin resistance plays a central role in the pathophysiology of Metabolic Syndrome (MS). Evidence indicates that MS begins with excess central adiposity [4]. In the genetically predisposed individuals, defects in insulin secretion follows, leading to impaired fasting glucose (IFG) and/or impaired glucose tolerance/glucose intolerance (IGT) [5]. Metabolic syndrome appears to have a component of heritability, which suggests a genetic basis [6]. Patients with rare single-gene disorders express the clusters of metabolic abnormalities associated with MS [6]. However, the association is complex and unresolved issues such as the role of gene-environment interactions, ethnicity, and gender in pathogenesis need to be further explored.

South Asians represent one-fifth of the global population and South Asian immigrants are the fastest growing immigrant population in many developed countries in the world. Studies have shown higher rates of CAD, insulin resistance and MS among South Asian immigrants living in developed countries [7]. The prevalence of CAD in South Asian immigrants was three times higher, even after adjustment for all conventional risk factors [8]. It is predicted that more than one-half of the world's CAD burden will be borne by people from the Indian subcontinent in coming decades [9]. Furthermore, evidence has shown that MS is twice more common amongst South Asian immigrants living in the US compared to the native Caucasian population [10]. Several studies have hinted at a possible genetic cause for this high prevalence of MS in South Asians [11]. The most recent surveys from Pakistan and India show that the prevalence of MS is 34.8% and 25.3% respectively [12]. However these surveys are either on hospital based samples or confined only to a specific regional locality of each country and presently there are no nationally representative studies on the prevalence of MS from any of the South Asian countries [12].

Sri Lanka is a middle income developing country in South Asia with a population of over 20 million. In 2005, the prevalence of hypertension and T2DM were nearly 20% and 11% respectively, while 1/5^th^ of the adult population were suffering from dysglysaemia (diabetes and pre-diabetes) [13,14]. In addition according to South-Asian cut-off values the prevalence of overweight (BMI ≥ 23 – 25.2%) and obesity (BMI ≥ 25 – 16.8%) were also at very high levels [15]. CAD (10.6%) is the leading causes of death in the country (by percentage of the total mortality for 2000) [16]. Presently there are no published studies on the prevalence of the metabolic syndrome in Sri Lankan. The present study aims to evaluate the prevalence of Metabolic Syndrome among Sri Lankan adults and investigates the relationships between MS and socio-demographic, clinical and biochemical parameters.

## Materials and methods

### Study population and sampling

Data on MS and its associated details were obtained from the Sri Lanka Diabetes and Cardiovascular Study (SLDCS). This population-based cross-sectional study was conducted in seven of the nine provinces in Sri Lanka between August 2005 and September 2006. The war affected Northern and Eastern provinces of the country were excluded from the study. Detailed sampling has been previously reported [13]. Relevant data from the nationally representative sample of 5000 non-institutionalized adults over 18 years of age are presented here.

### Measurements

Data collection was carried out by a field team of medical graduates and nurses who were trained in research methodology prior to commencement of data collection. Details of blood sample collection and biochemical analysis are described elsewhere [13]. Seated blood pressure was recorded on two occasions after at least a 10-min rest using an Omron IA2 digital blood pressure monitor (Omron Healthcare, Asia-Pacific Region, Singapore). Height was measured using Harpenden pocket stadiometers (Chasmors Ltd, London, UK) to the nearest 0.1 cm according to standard methods. Body weight was measured in indoor light clothing to the nearest 0.1 kg using a SALTER 920 digital weighing scale (Salter Ltd, Tonbridge, UK). Waist circumference was measured at midway between iliac crest and lower rib margin at the end of normal expiration using a plastic flexible tape to the nearest 0.1 cm. Similarly, the hip circumference was also measured at the widest part of the buttocks at inter-trochantric level to the nearest 0.1 cm. The study teams were regularly trained and evaluated on anthropometric measurements to minimize intra- and inter-observer variations. Each training session concluded with a standardization exercises, which included supervised measurement exercises repeated as many times as necessary until no large differences were observed between measurers.

Body Mass Index (BMI) was calculated as weight in kilograms divided by height in meters squared (kg/m^2^). An interviewer administrated questionnaire was used to obtain socio-demographic details such as age, gender, area of residence, ethnicity, level of education and household income. Physical activity during the past week was assessed using, the short version of the IPAQ (International Physical Activity Questionnaire) administered by an interviewer. Urban and rural sectors were defined according to the classification of the Sri Lankan government. The study was approved by the Ethics Review Committee of the Faculty of Medicine, University of Colombo, Sri Lanka.

### Definitions

This report is based on the definition of Metabolic Syndrome according to the International Diabetes Federation (IDF) criteria given below [17]:

(1) Raised Triglycerides >150 mg/l (1.7 mmol/l) or specific treatment for hypertriglyceridaemia.

(2) Low HDL-cholesterol < 40 mg/l (1.03 mmol/l) in males and < 50 mg/l (1.29 mmol/l) in females or specific treatment for low HDL-cholesterol.

(3) Raised blood pressure: systolic blood pressure > 130 mmHg or diastolic blood pressure > 85 mmHg or treatment for previously diagnosed hypertension.

(4) Dysglycaemia: fasting plasma glucose > 100 mg/l (5.6 mmol/l) and/or 2 h post-oral glucose tolerance test glucose >7·8 mmol/l or previously diagnosed type-2 diabetes.

Those with the presence of central obesity together with any two of the above parameters were classified as having MS. Central obesity was classified as waist circumference > 90 cm for males and > 80 cm for females. For the purpose of comparison with other regional and worldwide data, the prevalence of MS is also reported based on other commonly used diagnostic criteria published by the; World Health Organization (WHO) and US National Cholesterol Education Program (NCEP-ATP III) [18,19].

### Statistical analyses

All data were double-entered and cross checked for consistency. Data were analysed using SPSS version 14 (SPSS Inc., Chicago, IL, USA) and Stata/SE 10.0 (Stata Corporation, College Station, TX, USA) statistical software packages. The significance of the differences between proportions (%) and means were tested using z-test and Student’s *t*-test or ANOVA respectively. Direct standardization of prevalence was performed according to the population data published by the United Nations population division and for the ‘WHO new world population’ [20]. A binary logistic regression analysis was performed using the dichotomous variable Metabolic Syndrome (0 = absent, 1 = present). The independent co-variants (reference category) included in the binary logistic regression analysis were; gender (‘male’), age category (‘ < 30’ years), area of residence (‘rural’), ethnicity (‘Tamil’), level of education (‘no formal education’), monthly household income (‘<LKR7,000’) and physical activity (‘active’). For each independent variable with more than two categories dummy variables were created. The first category was taken as the reference category. A similar binary logistic regression analysis with above dependant and independent variables was also performed separately for both males and females. In all statistical analyses *P* values < 0.05 were considered significant.

## Results

Sample size was 4,485 (Response rate – 89.7%), 39.5% (n = 1,772) were males. Mean age was 46.1 ± 15.1 years (range: 18–90 years). Socio-demographic data of the study population is summarized in Table 1. Data from 4388 subjects aged ≥20 years were used for prevalence estimations. The crude prevalence of MS was 27.1% (95% CI: 25.8 – 28.5), and age-adjusted prevalence was 24.3% (95% CI: 23.0 – 25.6) (IDF criteria). For the purpose of comparison with other regional and worldwide data, the prevalence of MS was also calculated based World Health Organization (WHO) and US National Cholesterol Education Program (NCEP-ATP III) criteria [18,19]. The age-adjusted prevalence of MS according to the WHO criteria in all adults, males and females were 34.4% (95% CI: 33.0 – 35.8), 31.3% (95% CI: 29.2 – 33.5) and 36.5% (95% CI: 34.7 – 38.3) respectively. Females had a significantly higher prevalence than males (p < 0.001). According to the NCEP-ATP III criteria the age-adjusted prevalence of MS in all adults was 23.0% (95% CI: 21.8 – 24.3). There was a no significant difference in prevalence between males (21.7% [95% CI: 19.8 – 23.7]) and females (23.9% [95% CI: 22.3 – 25.5]).

**Table 1:** Socio-demographic characteristics in all adults, males and females

	Number of participants (%)		
	All	Males	Females
Area of residence			
Urban	955 (21.3)	363 (20.5)	592 (21.8)
Rural	3,530 (78.7)	1,409 (79.5)	2,121 (78.2)
Age category			
< 30 years	740 (16.5)	312 (17.6)	428 (15.8)
30-39 years	887 (19.8)	333 (18.8)	554 (20.4)
40-49 years	1,090 (24.3)	418 (23.6)	672 (24.8)
50-59 years	896 (20.0)	347 (19.6)	549 (20.2)
60-69 years	537 (12.0)	212 (12.0)	325 (12.0)
> 70 years	335 (7.5)	150 (8.5)	185 (6.8)
Ethnicity			
Sinhalese	3,877 (86.4)	1,521 (85.8)	2,356 (86.8)
Tamil	299 (6.7)	134 (7.6)	165 (6.1)
Sri Lankan Moor	298 (6.6)	114 (6.4)	184 (6.8)
Level of Education			
No formal education	267 (6.0)	56 (3.2)	211 (7.8)
Primary education	809 (18.0)	306 (17.4)	503 (18.5)
Secondary education	3,279 (73.1)	1,334 (75.3)	1,945 (71.7)
Tertiary education	129 (2.9)	76 (4.3)	53 (2.0)
Monthly Household Income			
≤ LKR 6,999	2,504 (56.3)	862 (49.1)	1,642 (60.9)
LKR 7,000 – 24,999	1,692 (38.0)	764 (43.6)	928 (34.4)
LKR 25,000 – 49,999	207 (4.7)	104 (5.9)	103 (3.8)
≥ LKR 50,000	47 (1.1)	24 (1.4)	23 (0.9)

LKR – Sri Lankan Rupees.

The remainder of the report considers MS as defined by IDF criteria. Age-adjusted prevalence in males and females were 18.4% (95% CI: 16.5 – 20.3) and 28.3% (95% CI: 26.6 – 30.0) respectively, with a significantly higher prevalence in females (p < 0.001). Most of the subjects with MS had 4 components of the syndrome (47.4%), 38.5% had 3, and 14.1% had all 5 components. Hypertension (81.1%), reduced HDL (77.0%), and elevated triglycerides (55.1%) were the most common abnormalities in all adults. A similar pattern was observed independently in both males and females (data not shown). Urban adults had a significantly higher prevalence of MS than their rural counterparts and this was also observed independently in both males and females (Table 2). The mean age of those with MS (51.4 ± 13.2 years) was significantly higher than those without MS (44.2 ± 15.3 years) (p < 0.001). The prevalence of MS increased significantly with increasing age in all adults, males and females (Table 2). In the different ethnic groups, the highest prevalence of MS was observed in Sri Lankan Moors (Muslims), followed by Sinhalese and Tamils (Table 2). A similar pattern was also observed in both males and females (Table 2). The prevalence of individual components of the MS (except low HDL cholesterol) was also highest in Sri Lankan Moors (Additional File 1). In all adults, the highest prevalence of MS was observed in those with the highest level of education (‘tertiary education’) and monthly household income (‘≥ LKR 50,000’). This was also observed independently in males, but not in females (Table 2). Females had a significantly higher prevalence of MS compared to males across all; areas of residence, age groups (except <30 years), ethnicities (except Tamils) and levels of education (except tertiary education) (Table 2).

**Table 2:** Age-standardized prevalence of Metabolic Syndrome in all adults, males and females

	Prevalence (95% Confidence Interval)			P value*
	All	Males	Females	
Area of residence				
Urban	34.8 (31.8 – 37.9)	24.3 (19.9 – 28.9)	40.8 (36.9 – 44.9)	<0.001
Rural	21.6 (20.2 – 23.0)	16.6 (14.7 – 18.7)	24.7 (22.9 – 26.6)	<0.001
Age category				
< 30 years	7.9 (5.9 – 10.3)	6.4 (4.0 – 9.7)	7.9 (5.6 – 10.9)	NS
30-39 years	21.4 (18.8 – 24.3)	17.4 (13.5 – 21.9)	23.8 (20.3 – 27.6)	<0.05
40-49 years	29.1 (26.4 – 31.9)	19.4 (15.7 – 23.5)	35.1 (31.5 – 38.9)	<0.001
50-59 years	34.3 (31.2 – 37.5)	27.7 (23.0 – 32.7)	38.4 (34.4 – 42.6)	<0.01
60-69 years	38.4 (34.2 – 42.6)	28.8 (22.8 – 35.4)	44.6 (39.1 – 50.2)	<0.001
> 70 years	35.5 (30.4 – 40.9)	22.0 (15.6 – 29.5)	46.5 (39.1 – 54.0)	<0.001
Ethnicity				
Sinhalese	23.3 (21.9 – 24.7)	17.0 (15.2 – 19.0)	27.2 (25.4 – 29.0)	<0.001
Tamil	20.6 (16.2 – 25.9)	16.7 (10.6 – 23.8)	26.4 (20.1 – 34.1)	NS
Sri Lankan Moor	43.0 (37.2 – 48.9)	35.7 (27.2 – 45.5)	45.2 (37.8 – 52.6)	<0.05
Level of Education				
No formal education	22.1 (17.3 – 27.7)	4.6 (1.1 – 14.9)	26.3 (20.3 – 32.5)	<0.001
Primary education	18.3 (15.6 – 21.1)	10.1 (7.0 – 14.1)	24.0 (20.4 – 28.0)	<0.001
Secondary education	25.8 (24.3 – 27.4)	19.5 (17.4 – 21.7)	30.5 (28.4 – 32.6)	<0.001
Tertiary education	28.2 (20.4 – 36.5)	35.2 (24.9 – 47.3)	15.9 (6.8 – 27.6)	NS
Monthly Household Income				
≤ LKR 6,999	20.1 (18.5 – 21.7)	12.3 (10.2 – 14.7)	24.3 (22.2 – 26.4)	<0.001
LKR 7,000 – 24,999	29.1 (26.9 – 31.3)	23.6 (20.6 – 26.7)	33.7 (30.7 – 36.9)	<0.001
LKR 25,000 – 49,999	26.6 (20.7 – 33.1)	22.2 (14.6 – 31.3)	35.8 (26.7 – 46.0)	NS
≥ LKR 50,000	30.1 (17.3 – 44.9)	27.9 (12.6 – 51.0)	28.6 (13.2 – 52.9)	NS

* P values – Males vs. Females, BMI – Body Mass Index, NS – Not significant, LKR – Sri Lankan Rupees.

Those with MS had a significantly higher weight, BMI, waist circumference, hip circumference, waist:hip ratio, systolic and diastolic blood pressure, fasting blood glucose, 2-hr post prandial blood glucose, total and LDL cholesterol and triglycerides (Table 3). This was observed in both males and females independently (data not shown). The mean weekly total MET minutes of those with MS (3491) was significantly lower than those without MS (5,142). A similar pattern was observed in both males and females separately (data not shown). The prevalence of MS in the different physical activity categories of the IPAQ was as follows; ‘inactive’ – 38.8% (95% CI 34.5-43.2), ‘moderately active’ – 33.5% (95% CI 30.9-36.1) and ‘active’ – 21.1% (95% CI 19.6-22.7). To evaluate the relationship between smoking and alcohol consumption on MS females were excluded, since there were only 5 current-/former- female smokers (0.2%) and 18 current-/former- female drinkers (0.6%). The age-adjusted prevalence of MS in males who have never smoked, former smokers and current smokers were 20.4% (95% CI: 17.5 – 23.6), 17.2% (95% CI: 13.5 – 21.4) and 14.5% (95% CI: 11.9 – 17.4) respectively. Among the current smokers, those who smoked 2–9 cigarettes per day had the highest prevalence (17.3%) followed by those smoking ≤ 1 per day (13.0%) and ≥ 10 per day (12.3%) respectively (p-NS). In males, those who had never consumed alcohol had the highest prevalence of MS (12.8% [95% CI: 10.1 – 15.9]), followed by current drinkers (10.3% [95% CI: 8.3 – 12.6]) and former drinkers (6.3% [95% CI: 4.1 – 9.2]). Among current male drinkers, highest prevalence was found in those who drank > 21 units/week (22.2% [95% CI: 14.5 – 31.7]), followed by those drinking <7 units/week (19.0% [95% CI: 16.1 – 22.2]) and 7–21 units/week (18.1% [95% CI: 10.5 – 28.0]).

**Table 3:** Mean values of clinical and biochemical parameters in those with and without Metabolic Syndrome

	Mean (±SD)		
	Metabolic Syndrome	Non-Metabolic Syndrome	p
Weight (kg)	60.5 (±11.7)	50.5 (±10.3)	<0.001
Body mass index (kgm^-2^)	25.1 (±4.0)	20.5 (±3.6)	<0.001
Waist circumference (cm)	87.4 (±9.8)	73.6 (±10.1)	<0.001
Hip circumference (cm)	95.4 (±8.7)	86.6 (±8.0)	<0.001
Waist:Hip ratio	0.9 (±0.1)	0.8 (±0.1)	<0.001
Systolic blood pressure (mmHg)	139.2 (±18.9)	122.8 (±18.2)	<0.001
Diastolic blood pressure (mmHg)	82.1 (±10.6)	72.9 (±10.5)	<0.001
Fasting blood glucose (mg/dl)	105.4 (±38.6)	86.4 (±22.0)	<0.001
2-hr post prandial blood glucose (mg/dl)	146.8 (±72.3)	99.7 (±37.4)	<0.001
Total cholesterol (mg/dl)	220.3 (±43.0)	201.8 (±42.7)	<0.001
LDL cholesterol (mg/dl)	143.6 (±38.2)	132.5 (±36.9)	<0.001
HDL cholesterol (mg/dl)	42.6 (±7.8)	48.3 (±11.1)	<0.001
Triglycerides (mg/dl)	169.8 (±81.9)	104.1 (±49.9)	<0.001

The results of the binary logistic regression analysis in all adults using the dichotomous variable ‘Metabolic Syndrome’ (0 = absent, 1 = present) as the dependant factor and other independent variables are shown in Table 4. The overall model was statistically significant as determined by the likelihood ratio test (*χ*2 = 38.22, p < 0.05). The Cox & Snell R-Square and Nagelkerke R Square values were 0.126 and 0.184 respectively. The results indicate that female gender (OR: 1.7), increasing age, urban living (OR: 1.7), Sri Lankan Moor ethnicity (OR: 2.6), secondary (OR: 1.5) and tertiary level of education (OR: 2.3), household income LKR 7,000 – 24,999 (OR: 1.5) and >50,000 (OR: 2.1), and physical inactivity (OR: 1.6), all were associated with significantly increased risk of developing MS (Table 4). Similar results were seen independently in males, however urban residency, smoking and alcohol consumption were not significant risk factors (Table 4). In females, level of education was not a significant risk factor (Table 4).

**Table 4:** Binary logistic regression analysis in all adults, males and females

	Odds ratio (9CI)		
Co-variants (Reference category)	All	Male	Female
Female Gender	1.7 (1.3 – 2.2)^*^		
Age category (< 30 years)			
30 – 39 years	3.8 (2.7 – 5.2)^*^	3.3 (1.9 – 5.8)^*^	3.8 (2.5 – 5.8)^*^
40 – 49 years	6.0 (4.4 – 8.3)^*^	4.2 (2.4 – 7.1)^*^	7.3 (4.9 – 10.9)^*^
50 – 59 years	8.1 (5.8 – 11.1)^*^	6.9 (4.0 – 11.8)^*^	9.5 (6.3 – 14.2)^*^
60 – 69 years	10.6 (7.5 – 14.9)^*^	9.0 (5.0 – 16.0)^*^	13.0 (8.4 – 20.1)^*^
> 70 years	8.3 (5.6 – 12.2)^*^	4.8 (2.5 – 9.2)^*^	13.2 (8.1 – 21.7)^*^
Urban residence	1.7 (1.4 – 2.0)^*^	1.1 (0.8 – 1.5)	1.8 (1.5 – 2.3)^*^
Ethnicity (Tamil)			
Sinhalese	1.2 (0.8 – 1.6)	0.5 (0.1 – 0.7)	1.4 (0.9 – 2.1)
Sri Lankan Moor	2.6 (1.7 – 3.8)^*^	1.8 (0.9 – 3.5)^*^	3.1 (1.9 – 5.1)^*^
Level of education (no formal education)			
Primary education	0.9 (0.7 – 1.3)	2.4 (0.8 – 7.1)	0.2 (0.02 – 2.2)
Secondary education	1.5 (1.1 – 1.9)^§^	3.4 (1.2 – 9.9)^§^	0.8 (0.6 – 1.1)
Tertiary education	2.3 (1.8 – 2.3)^*^	4.9 (1.5 – 16.0)^#^	1.1 (0.8 – 1.6)
Monthly Household Income (≤ LKR 6,999)			
LKR 7,000 – 24,999	1.5 (1.3 – 1.8)^*^	1.7 (1.3 – 2.3)^*^	1.4 (1.2 – 1.8)^*^
LKR 25,000 – 49,999	1.2 (0.8 – 1.7)	1.2 (0.7 – 2.1)	1.2 (0.8 – 2.0)
≥ LKR 50,000	2.1 (1.1 – 4.2)^*^	2.2 (0.8 – 5.7)	2.2 (0.9 – 5.6)
Physical inactivity	1.6 (1.3 - 1.9)^*^	1.9 (1.3 – 2.7)^*^	1.5 (1.2 – 1.8)^*^
Smoking (non-smokers)			
Former smokers	1.1 (0.8 – 1.5)	1.2 (0.8 – 1.7)	
Current smokers	0.7 (0.5 – 0.9)	0.8 (0.6 – 1.1)	
Alcohol consumption (non-consumers)			
Former drinkers	0.6 (0.4 – 0.8)^§^	0.7 (0.4 – 1.0)	
Current drinkers	0.9 (0.7 – 1.3)	0.9 (0.7 – 1.3)	

* - p < 0.001, # - p < 0.01, § - p <0.05.

## Discussion

Metabolic Syndrome is known to increase the risks of Coronary Artery Disease (CAD), stroke, and type-2 diabetes [1,2]. Recent surveys from Pakistan and India show that the prevalence of MS is 34.8% and 25.3% respectively [12]. However these surveys are either on hospital based samples or confined only to specific regional localities [21]. This is the first report on the prevalence of MS among ethnic South Asian adults based on a community-recruited, nationally representative sample from a South Asian country [12]. In addition, higher sample size and response rate were the main strengths of the study, however there was a female predominance in the study cohort which could have been due to the fact that the study was conducted during the day time. In most South Asian countries females are usually housewives while the males engage in work. We report an age-adjusted prevalence of 24.3% in all adults (males: 18.4%, female: 28.3%). The prevalence is similar to that of other reports from regional South Asian countries such as; India (Southern India – 25.8%, Northern India – 25.3%), Pakistan (27.0%) and Nepal (22.5%) [22-24]. These studies have also used the IDF criteria for diagnosis, and they have been conducted during a similar time period to the present study. However, they have been confined to only a specific locality in each country. In contrast, our prevalence and the prevalence in South Asian countries are much higher than that of other regional Asian countries such as; Singapore (17.7%), Taiwan (13.9%), Japan (7.8%) and China (18.3%) [25-28]. Prevalence of MS in USA (39.0%), Greece (43.4%), and western developed counties are much higher than the prevalence in developing South Asian countries [29,30]. However, evidence has shown that MS is twice more common amongst South Asian immigrants living in the US compared to the native Caucasian population [10]. Hence, it is possible to postulate that adoption of western lifestyles after migration has led to the increased prevalence of MS amongst immigrant South Asians.

The present study used the definition proposed by the IDF for the purpose of defining MS. The IDF definition of MS was developed in the light of the confusion, ambiguity and limitation created by other definitions. Especially amongst Asians the likely inappropriateness of other definitions such as the NCEP-ATPIII has been highlighted by data showing that the application of this definition to Asian populations results in a very low prevalence of the metabolic syndrome, which is out of keeping with the high prevalence of diabetes in such populations [31]. This is due to the inappropriateness of the NCEP-ATPIII waist circumference cut-points for non-Europid populations. Current evidence suggests that the WHO definition is better able to predict those at risk of CVD than the NCEP-ATPIII, a feature which has been attributed by some to the central position of insulin resistance in the WHO construct [32]. Furthermore, when insulin resistance was added to the NCEP-ATPIII, it performed similar to the WHO definition [32]. However, measurement difficulties have limited the inclusion and usefulness of insulin resistance in the definition. Hence, the definition developed by the IDF consensus group finds some compromise between the WHO and the NCEP-ATPIII definitions, while addressing ethnic differences between populations [33].

The reasons for the high prevalence of MS among Sri Lankan adults are multi-factorial. As in other South Asian countries Urbanization and mechanization in Sri Lanka have led to a sedentary life style and changes in dietary patterns [12]. Several studies have also reported the role of genetics in the development of obesity and MS in South Asians [12,34]. Among South Asian immigrants in USA three novel Apo lipoprotein A-1 single nucleotide polymorphisms were found to be significantly associated with the presence of MS [34]. However, further studies are required to establish a cause-effect relationship and identify genetic factors that are responsible. In the present study, increasing age, female gender, urban residency, Sri Lankan Moor ethnicity and physical inactivity were significant risk factors associated with MS among Sri Lankan adults. Age dependency in the prevalence of MS is observed in most populations around the world [35]. Our results show that prevalence of MS increased with age, while increasing age was the strongest risk factor associated with MS with an OR of 13.2 in females (>70 years) and OR of 9.0 in males (60–69 years). Substantial decline in both fertility and mortality rates in Sri Lanka has led to an unprecedented increase in the aging of its population [36]. It is estimated that the geriatric population in Sri Lanka in the coming decades would consist of a higher proportion of females [11]. Our results show that female gender was also a significant risk-factor and the association between age and MS was even stronger amongst females. The significantly higher prevalence of MS among females could also be partly due to using a lower WC cut-offs for defining abdominal obesity. Regional studies advocate the use of same cut-off values for both genders in South Asians [37]. Furthermore, both age and gender are non-modifiable risk factor and what is important is to recognize these high vulnerable groups and institute selective preventive measures. Urban residency was also significantly associated with the prevalence of MS among Sri Lankan adults. The association between MS and urban living is well-established, especially in the developing South Asian countries [12]. Globalization of diets and consumption of non-traditional foods have occurred at a rapid pace in urban areas, and alarmingly these dietary changes are more common amongst the children and younger adults [38,39]. The rapid increase in fast food outlets, sale of aerated sweet drinks, and increased consumption of fried snacks are common in urban areas [39]. It is also well known that South Asians are less physically active than other ethnic groups [40]. Unhealthy dietary habits, physical inactivity and stress, all combine to propagate the increasing occurrence of MS among urban adults [41].

Higher socio-economical status as defined by education and income were associated with increased prevalence of MS among Sri Lankan adults. The relationship between educational level and MS is less distinct amongst females due to lower numbers in higher educational categories. Furthermore, although higher income was associated with an increase in MS in all adults, it is not clearly evident when genders are considered separately. This could be due to considering household rather than individual income in the present study. This direct association between MS and higher social class could be due to the increased ability to purchase food in those with a higher socio-economical status. A similar trend was observed between obesity and social class in Tunisia, a middle-income developing country, where the increased ability to purchase food was believed to increase the calorie intake and hence obesity [42]. However, these findings are in contrast to affluent countries, where obesity and MS are associated with lower income and socio-economical status [43,44]. Among the different ethnicities Sri Lankan Moors had a higher prevalence of MS and the Sri Lankan Moor ethnicity was a significant risk-factor associated with MS in the regression analysis. This could be due to differential dietary habits and physical activity patterns among the different ethnicities. Hence, it is important to consider ethnic differences when instituting preventive measures.

Cigarette smoking is known to be independently associated with metabolic syndrome, and demonstrate a dose–response relationship [34]. The relationship between alcohol consumption and MS is less distinct. Some studies report a possible association between alcohol intake and risk of MS [45]. Other reports have demonstrated a beneficial effect of alcohol intake on the risk of MS, suggesting that this relationship is rather complex [46]. Alcohol consumption was reported to have favorable effects on plasma HDL cholesterol and insulin sensitivity, as well as unfavorable effects of increase in plasma triglycerides and blood pressure [47]. Our results indicate that prevalence of MS is higher among non-smokers than smokers; however smoking was not a significant risk-factor in the logistic regression analysis. Hence the relationship between smoking and MS is not as distinct in the present study as previously reported [34]. However, moderate alcohol consumption (7–21 units per week) was associated with a lower prevalence than those consuming higher amounts. In the present study cigarette smoking and alcohol consumption were self-rated; objective measures confirming these self-reported data, such as measuring nicotinine in blood, urine, or saliva, could have increased the validity of the data. However, such measures would have not been feasible in the context of the present study. Diet plays an important role in the pathogenesis of MS. A whole array of dietary factors, such as unbalanced fat consumption, high sodium intake, low dietary fiber and unhealthy eating patterns are reported to contribute to development of obesity, diabetes and cardiovascular disease among South Asians [41]. Nutrition in relation to the MS has been poorly researched among South Asians including Sri Lankans [12]. Sri Lankans consume higher amounts of carbohydrates, which fulfill around 70% total daily energy intake (Unpublished data). The consumption of large carbohydrate meals may cause postprandial hyperglycemia and hypertriglyceridaemia [41]. Presently there is a lack of data on diet and association with MS risk factors among Sri Lankan adults and further studies exploring this association are required.

The value in the diagnosis of MS in an individual is an area of much debate. Studies have shown an increased CAD risk among subjects with 1 or 2 risk factors not falling under the MS definition, suggesting that irrespective of the presence or absence of MS all risk factors should be managed aggressively [48]. Among the native Chinese population all MS criteria (IDF, WHO, NCEP) failed to predict CAD but predicted the occurrence of diabetes [49]. MS criteria are known to be inferior to the Framingham score for prediction of vascular events [50]. Although MS has several limitations on predicting vascular events, the concept of MS strengthens the clinical interest and understanding of a group of metabolic parameters among different medical experts such as cardiology, vascular physicians, and endocrinologist [50]. Importantly, because of the MS concept all physicians emphasize the importance of lifestyle changes, in particularly weight reduction, increase physical activity and healthy eating. The strengths of the present study are its’ national representativeness, random selection of subjects out of a well-defined and homogenous target population and the sizes of the sample population groups with a high response rate. The inability to include subjects from the Northern and Eastern provinces of the country due to the war is a limitation of the present study. Majority of the Tamils in Sri Lanka reside in these two provinces, while the Tamils included in the present study were mainly plantation workers of Indian origin. Furthermore the cross-sectional design of our study limits the inference of causality for the risk factors identified. Therefore, it is important to conduct prospective studies on MS and look for causality for both CAD and diabetes. In addition although diet has a direct effect on MS we were unable to evaluate dietary habits of Sri Lankan adults during the present survey, due to the lack of a validated dietary assessments tool and a nutrition composition database on local mixed dishes. However, currently we are in process of developing a country specific Food Frequency Questionnaire and nutrition composition database.

## Conclusions

Metabolic Syndrome is common among Sri Lankan adults affecting nearly one-fourth of the adult population. Female gender, increasing age, urban living, higher socio-economical status and physical inactivity were all important factors associated with the occurrence of Metabolic Syndrome. It is important to institute holistic, multidisciplinary, and multi-sectoral preventive measures at an individual, community, and societal level focusing on promoting healthy dietary habits and physically active life-styles to fight the growing epidemic of Metabolic Syndrome in Sri Lanka.

## Competing interests

The author(s) declare that they have no competing interests.

## Authors’ contributions

PK, DRM and MHRS made substantial contribution to conception and study design. PK, PR and RJ were involved in data collection. RJ and PR were involved in refining the study design, statistical analysis and drafting the manuscript. RJ, PR, MHRS and PK critically revised the manuscript. All authors read and approved the final manuscript.

## Supplementary Material

Additional file 1 The prevalence of individual components of Metabolic Syndrome in different ethnicities. Click here for file
