# Supplementary material for: Metabolic syndrome among Sri Lankan adults: prevalence, patterns and correlates
Source: Diabetol Metab Syndr. 2012 May 31;4:24. doi: 10.1186/1758-5996-4-24 (PMC3407762; doi:10.1186/1758-5996-4-24)
Supplement: Additional file 1 — The prevalence of individual components of Metabolic Syndrome in different ethnicities. [file 1758-5996-4-24-S1.doc]

The prevalence of individual components of Metabolic Syndrome in different ethnicities

|  | Prevalence (95% CI) | | |
| --- | --- | --- | --- |
|  | Sinhalese | Tamils | Sri Lankan Moor |
| Central Obesity  ( M: > 90 cm, F: > 80 cm) | 29.8 (28.3 – 31.3) | 17.7 (13.6 – 22.5) | 48.3 (42.5 – 54.2) |
| Triglycerides*  (> 150 mg/l) | 22.1 (20.8 – 23.4) | 20.7 (16.3 – 25.8) | 35.6 (30.1 – 41.3) |
| HDL-c  (M: < 40 mg/l, F: < 50 mg/l) | 47.7 (46.2 – 49.3) | 61.9 (56.1 – 67.4) | 56.4 (50.5 – 62.1) |
| Blood pressure*  (SBP > 130 mmHg, DBP > 85 mmHg) | 42.5 (41.0 – 44.1) | 37.8 (32.3 – 43.6) | 50.3 (44.5 – 56.2) |
| Blood Sugar*  (FPG > 100 mg/l, 2-hr OGTT > 140 mg/l) | 18.1 (16.7 – 19.3) | 15.7 (11.8 – 20.4) | 29.2 (24.1 – 34.7) |

* or being on specific treatment for either hypertriglyceridaemia, hypertension or diabetes mellitus; M – Males, F – Females, HDL-c – HDL-cholesterol, FPG – Fasting Plasma Glucose, OGTT – Oral Glucose Tolerance Test
